# Towards Multimodal Machine Learning Prediction of Individual Cognitive Evolution in Multiple Sclerosis

**DOI:** 10.3390/jpm11121349

**Published:** 2021-12-11

**Authors:** Stijn Denissen, Oliver Y. Chén, Johan De Mey, Maarten De Vos, Jeroen Van Schependom, Diana Maria Sima, Guy Nagels

**Affiliations:** 1 AIMS Laboratory, Center for Neurosciences, UZ Brussel, Vrije Universiteit Brussel, 1050 Brussels, Belgium; johan.de.mey@vub.be (J.D.M.); jeroen.van.schependom@vub.be (J.V.S.); diana.sima@icometrix.com (D.M.S.); guy.nagels@vub.be (G.N.); 2 icometrix, 3012 Leuven, Belgium; 3 Faculty of Social Sciences and Law, University of Bristol, Bristol BS8 1QU, UK; olivery.chen@bristol.ac.uk; 4 Department of Engineering, University of Oxford, Oxford OX1 3PJ, UK; 5 Department of Radiology, UZ Brussel, Vrije Universiteit Brussel, 1090 Brussels, Belgium; 6 Faculty of Engineering Science, KU Leuven, 3001 Leuven, Belgium; maarten.devos@esat.kuleuven.be; 7 Faculty of Medicine, KU Leuven, 3001 Leuven, Belgium; 8 Department of Electronics and Informatics (ETRO), Vrije Universiteit Brussel, 1050 Brussels, Belgium; 9 St Edmund Hall, Queen’s Ln, Oxford OX1 4AR, UK

**Keywords:** multiple sclerosis, prognosis, cognition, machine learning, artificial intelligence

## Abstract

Multiple sclerosis (MS) manifests heterogeneously among persons suffering from it, making its disease course highly challenging to predict. At present, prognosis mostly relies on biomarkers that are unable to predict disease course on an individual level. Machine learning is a promising technique, both in terms of its ability to combine multimodal data and through the capability of making personalized predictions. However, most investigations on machine learning for prognosis in MS were geared towards predicting physical deterioration, while cognitive deterioration, although prevalent and burdensome, remained largely overlooked. This review aims to boost the field of machine learning for cognitive prognosis in MS by means of an introduction to machine learning and its pitfalls, an overview of important elements for study design, and an overview of the current literature on cognitive prognosis in MS using machine learning. Furthermore, the review discusses new trends in the field of machine learning that might be adopted for future studies in the field.

## 1. Introduction

As one of the most puzzling neurodegenerative disorders, multiple sclerosis (MS) is characterized by a complex biological etiology [1] and a highly heterogeneous disability progression. This gives rise to an important unmet need that has been given considerable attention in MS research in recent decades, which is the prediction of its future course [2,3,4,5]. In light of an ongoing paradigm shift in medicine, moving from a disease-centered to a patient-centered approach [6], the ability to foresee disability build-up in a specific patient would be a true game changer in modern medicine; neurologists could intervene at an early stage, whereas patients and their caregivers could anticipate future challenges in daily life.

Currently however, to predict the natural course of MS on an individual level remains challenging. Foremost, the problem is intrinsically difficult since the disease manifests differently among patients. From a biological point of view, tissue damage in the central nervous system (CNS), caused by auto-immune processes, is not restricted to a single location or to a particular timepoint during the disease course [7]. Typical observations are the presence of lesions, resulting from processes such as demyelination and inflammation, in conjunction with the loss of CNS tissue [7]. However, MS patients typically present a wide range of clinical symptoms as well, ranging from motor and sensory impairments to fatigue, cognitive problems, and mental health issues [8]. Since every person with MS presents a unique biological and clinical profile, health-related predictions should be individualized.

At present, the best tools to estimate individual disease progression are the so-called prognostic biomarkers. They are defined by Ziemssen et al., 2019, as: “A prognostic biomarker” that “helps to indicate how a disease may develop in an individual when a disorder is already diagnosed” [9]. Although these variables can be regarded as the cobblestones of the road towards an accurate prognostic model, it is important to note that this term is assigned regardless of any magnitude of prognostic accuracy. Moreover, they are typically established at group level, which might be a suboptimal fit in light of the aforementioned heterogeneity across subjects with MS.

In a recent systematic review by Brown et al., 2020, the authors identified several studies that used various statistical techniques to combine prognostic biomarkers [2]. Although the techniques used are widespread, some studies report on the use of machine learning (ML), allowing personalized predictions of the behavior of a clinically relevant variable over time. The literature on this topic was synthesized by Seccia et al., 2021, although the authors limited their search to models using clinical data [4]. As can be expected from a young field of research, a sprawl of underlying methodology is observed among papers that use ML to perform prognostic modelling in MS; heterogeneity in terms of input features, learning algorithms, labels to predict, and assessment metrics hamper comparability among models. The narrative nature of both aforementioned reviews underscores the fact that quantitative synthesis by means of, e.g., meta-analysis or meta-regression, is not yet possible. Furthermore, various models aim to predict disease progression in terms of changes in the Expanded Disability Status Scale (EDSS), while a recent review by Weinstock-Guttmann et al., 2021, questions the use of the EDSS for prognostic purposes due to a lack of accuracy and stability [3]. This review also highlights the importance to look at other domains, such as cognitive impairment [3]. Problems in various cognitive domains are prevalent in persons with MS, especially in memory and information processing speed [10]. Since cognitive functioning was shown to be related to socio-economic aspects such as employment status [11] and income [12], prognostication in this domain could allow patients and their caregivers to anticipate future problems at an early stage.

Although the use of machine learning for cognitive prognosis is still in its infancy, this paper aims to offer directions in this field by (1) introducing the concept of machine learning, (2) outlining the pitfalls of machine learning in medical sciences, (3) offering guidance for the design of studies that use ML for cognitive prognosis using lessons learned from ML-powered physical prognosis, (4) summarizing literature on ML-powered cognitive prognostication, and (5) highlighting trends in ML that could boost the field of MS prognosis. Since the main goal of this review is to provide directions for a young field of research rather than to synthesize the scarcely available literature, this review adopts a narrative, non-systematic design.

## 2. An Introduction to Machine Learning

Machine learning is defined in the Oxford University Press (OUP) as: “The use and development of computer systems that are able to learn and adapt without following explicit instructions, by using algorithms and statistical models to analyse and draw inferences from patterns in data” [13]. Although learning and adaptation can happen in multiple ways, typically categorized as “supervised”, “unsupervised” and “reinforcement” learning, the most common machine learning technique adopted in the medical sciences is supervised machine learning. The notion of “supervision” here is the presence of the ground-truth label to be predicted, which can either be a continuous variable (regression) or a categorical variable (classification). In general, the goal is to learn the relationship, in terms of a function, between a given input and the output—the ground-truth label. The function that subsequently best predicts the ground-truth label on input data that was not used to learn the function is the model of choice.

The concept can be clarified by means of an analogy; a student studying for a future exam. In the first phase, the student will gather knowledge on the domain by using available resources such as books and lecture notes (training). The student subsequently verifies whether additional study is necessary by completing an exam from previous years to which the answers are available (validation). Together, this is called the training phase. As necessary, training and validation are repeated until the student is ready to take the final exam, which constitutes the testing phase.

Let us assume that we want to use supervised machine learning to predict a person’s age given a brain magnetic resonance (MR) image. We start from a dataset with T1-weighted brain MR images (input) and the age at image acquisition (ground-truth label). Since age is a continuous variable, we are facing a regression problem. How we will learn the relationship between MRI and age depends on how we will use the MRI:

- Classical approach. The first approach is to analyze the brain MR images, yielding a set of features that describe the image such as volumetric quantifications of brain structures. This allows for the use of more classical supervised learning algorithms such as linear/logistic regression, support vector machines (SVM) and random forests (RF). Table 1 summarizes some frequently used supervised learning algorithms;
- Deep learning. The second option is to use the raw brain MR images as input and use a technique called deep learning, which recently gained popularity as a subtype of machine learning. The major difference compared to classical machine learning is that it mitigates the necessity to manually transform raw data in a meaningful feature representation, the so-called “feature engineering” step, relying on human domain-specific knowledge [14]. Deep learning will automatically create meaningful representations from raw data, thus achieving representation learning [14]. This will typically yield “latent features”, which are hard to interpret by humans, but are deemed by the machine to be relevant. The advantage of deep learning lies in the more complex relationships that can be learned, while a major drawback is the need for large datasets, time and computational power.

## 3. Caveats for Machine Learning and Potential Solutions

Numerous pitfalls can be encountered when performing machine learning. The majority of them are generally applicable; they could arise in any machine learning query in any domain. Yet, we can encounter hazards that are specific for medical sciences. Both are discussed in this section, and solutions used in the field of prognostic modelling are also summarized.

### 3.1. General Pitfalls in Machine Learning

**Figure 1:**
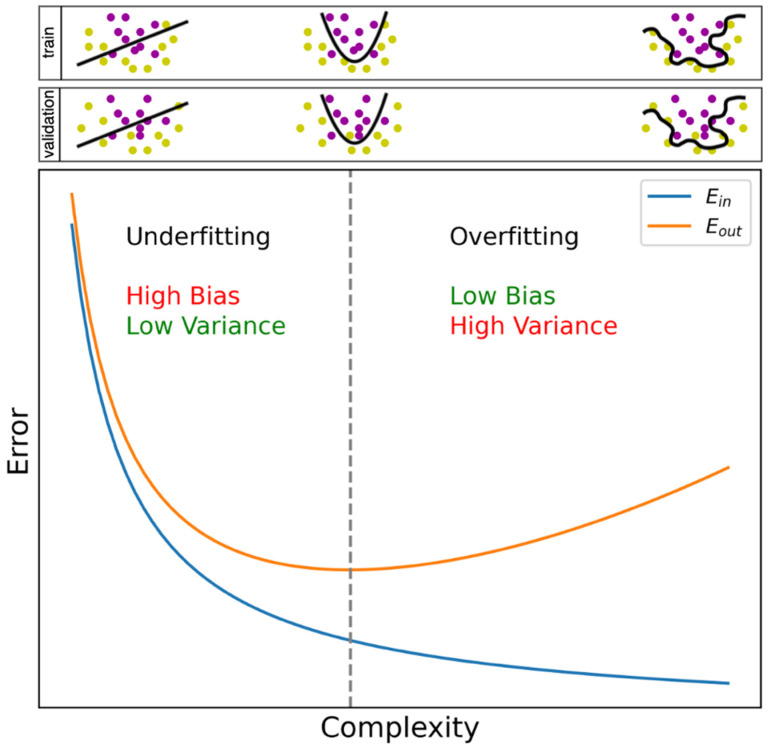
Bias–variance trade-off curve. Bias and variance vary according to model complexity [16]. The blue curve is E_in_, the within-sample error representing the error on the training dataset. The more complex a function is allowed to be, the more specific the function becomes for the training dataset, i.e., overfitting. The latter is notable by the inception of an increase in E_out_ (orange curve, minimal value indicated with the vertical dotted line), the out-of-sample error, representing the error on the validation dataset. A simple function suffers high bias, i.e., it is highly likely to assume a wrong underlying function, since it only allows limited complexity between input and output to be learned (underfitting). By allowing more complexity, the bias decreases, but the function becomes highly variable depending on the dataset used for training (overfitting). An illustration is provided above, where the learned function is the line or curve separating two classes. From visual inspection, the optimal situation would be a smooth curve between the two classes (example in the middle). In the example on the left, underfitting occurs since only a straight line is allowed; many misclassifications occur in both training and validation data. In the example on the right, we observe a curve that squirms around all datapoints to fit the training dataset (overfitting), which, for example, happens when we allow the model to learn a complex function capable of learning measurement errors in a dataset. Hence, the function becomes specific to the training dataset; no misclassifications occur in the training data, but the same curve separating the validation dataset yields many misclassifications.

The most common pitfall in any machine learning query is overfitting. As already mentioned, a function is learned on training data and evaluated on validation and test data. Overfitting means that our learned function has become very specific to the training data, for example, because it also learned measurement errors in that dataset. Since measurement errors are different in another dataset, the function will be less accurate on that dataset. It is also possible, however, that we underestimate the complexity of the problem, which is the exact opposite case and understandably termed underfitting. For example, linear regression assumes a linear relationship between input features and the endpoint, which limits the model to only learn linear relationships, while the problem might be non-linear in reality. Figure 1 serves as a visual aid towards the understanding of under- and overfitting.

Overfitting often results from an imbalance between the number of variables and observations in the dataset. As a rule of thumb in the field, the number of observations should be at least 10 times as high as the number of variables [17]. To get to that ratio, we can address an imbalance in two ways: upscaling the observations or downscaling the variables. We note that in the case of downscaling variables, one should always remain vigilant not to underfit; informative features might be rejected as well.

1. Addressing the observations. Upscaling the number of observations is one way of tackling overfitting, but researchers often possess a database with a fixed number of observations. Nonetheless, several techniques exist to increase the number of observations based on those already present, e.g., using data augmentation. Although numerous variants exist, an easy-to-grasp data augmentation method is the insertion of random noise into an observation [18], and can be interpreted as a similar, yet different subject record. A generative adversarial network (GAN) [19] serves the same purpose, which we will explain by means of a metaphor. Imagine a game-like situation in which a radiologist has to find out whether an image is a true MR image of the brain or was produced by a computer, i.e., a “villain”, trying to fool the radiologist. Initially, the radiologist will easily identify which images were produced by the villain, since it had no clue how to generate a representative image. However, since the villain receives feedback on its effort, it will gradually start to understand how to create an image that will give the radiologist a hard time in telling whether it is a true image or a fake one. The radiologist on the other hand is forced to keep on improving classification skills, since it gradually becomes harder to distinguish them, in turn stimulating the villain to propose better images. Hence, the radiologist and the villain will infinitely stimulate each other to perform better. Ultimately, MR images are produced by the villain that could in fact have been the true ones, and which can subsequently be used to expand a dataset. Similar to deep learning, a GAN needs, besides time and computational power, large amounts and diversity of data to create qualitative new observations;
2. Addressing the features. The second option to restore an imbalance is the reduction of the number of features that the algorithm will be trained on. In feature selection, only the features that are deemed informative are selected. For an outline of several feature selection techniques in the context of medical sciences, we refer to Remeseiro et al., 2019 [20]. The original set of features can also be transformed to a new set of features. This can, for example, be done with principal component analysis (PCA), where we could say that the features are “reordered”; an equal number of features are obtained—the “principal components”—that explain variance in the data in a decreasing order. Feature selection can then occur on principal components instead of the original features. The additional benefit of PCA is that it is a solution to the problem of multi-collinearity, in which features are mutually correlated. As a result, two variables might contain similar information, while the resulting principal components from PCA are uncorrelated [21].

Besides addressing observations and features, we discuss one additional technique to mitigate overfitting, which is training interruption. In their efforts to predict the progression of disease, Bejarano et al., 2011 [22] and Yoo et al., 2016 [23] stopped the training phase early by monitoring the error in the validation set. As can be seen in Figure 1, the error in the training set keeps reducing over time, since this is the goal of training. Initially, the same is observed for the validation data set, but upon obtaining a minimal value, the error will gradually increase, indicating the inception of overfitting. When stopping training at this point, overfitting might be mitigated.

Class imbalance is a specific pitfall for classification problems and is present when a certain class is overrepresented in the data, i.e., it contains more observations compared to the other class(es). For prognosis, subjects that do not worsen over time are often in the majority compared to worsening subjects [24,25]. Like overfitting, it can lead to the poor generalization of an algorithm [26]. Methods to correct class imbalance in a deep learning context are summarized in a systematic review by Buda et al., 2018 [26]. Two types of corrections are discussed, addressing either the data or the classifier itself. When addressing the data, we could restore the balance in two ways: by oversampling the minority class or by undersampling the majority class. On the other hand, we can make adjustments when training or testing the classifier. For example, one could decide to more severely penalize a misclassification towards a certain class compared to a misclassification towards another class, i.e., cost-sensitive learning [26]. These three methods were already explored in the light of prognostic modelling in MS to address the imbalance between stabilizing and worsening subjects [24,25].

### 3.2. Specific Pitfalls for Medical Data

Next to several general pitfalls, there are additional pitfalls when working with medical data:

1. Study data versus real-world data. Although the standardization of conditions and minimizing missing values are in general considered good practice, for example, when collecting data as part of a research study, it might limit the use of models in daily clinical routine that are known to be contaminated with, e.g., measurement errors, non-standardized test intervals, and missing values. When an algorithm encounters such inconsistencies during training, it could be expected to perform better on out-of-sample data. Although well-curated study data still dominate the field of prognostic modelling, efforts are underway to expand the use of real-world data [27,28];
2. Single-center versus multi-center data. This argument is similar to the former; data from different clinical centers might be different due to discrepancies in testing equipment (e.g., MRI scanner), testing protocols, and patient characteristics. Introducing this heterogeneity already during the training phase might increase generalizability;
3. Multiple visits of the same patient. Finally, when using multiple visits of a patient as separate observations in a dataset, one should always remain vigilant that the visits do not get intermingled between train, validation, and test datasets. Since visits are often highly comparable, the performance on an unseen test dataset could be biased, performing better than would be the case when adopting a truly independent test dataset. This could be categorized under the hazard called “leakage”, in which information of the test dataset leaks in the training dataset. To prevent this from occurring, Seccia et al., 2020 applied a correctional method called “leave one group out” (LOGO) [27]. With this method, they withdrew all visits of one subject from the training set and used them as a test set, after which the procedure was repeated for all subjects. This hinders models to recognize patients within a dataset. Other methods were, for example, discussed in Tacchella et al., 2018 [29] and Yperman et al., 2020 [28].

## 4. Designing an ML Study for Cognitive Prognosis

Supervised machine learning is popular for its ability to provide personalized predictions on health parameters that clinicians are used to work with in routine practice. One of these use cases includes predictions on how a patient with a certain condition progresses over time (prognosis) [30]. In the following section, we will address relevant questions when designing a machine learning study for cognitive prognosis in MS, using a question and answer (Q&A) approach. Answers are mostly constructed using lessons learned from the literature on ML-powered physical prognosis in MS and the literature on cognitive prognostic biomarkers.

### 4.1. Which Outcome to Predict?

As mentioned before, the outcome (categorical versus continuous) will define the type of problem we are facing: classification versus regression. When looking at cognitive outcomes, the most commonly affected domains are information processing speed and memory [10]. According to Sumowski et al., 2018, information processing speed is best assessed with the Symbol Digit Modalities Test (SDMT), whereas for memory, the brief Visuospatial Memory Test—Revised (BVMT-R), California Verbal Learning Test—Second Edition (CVLT-II), and Selective Reminding Test (SRT) are the most sensitive tests [31]. However, composite scores also exist to provide a more holistic view on the cognitive status of persons with MS, which are summarized in Oreja-Guevara et al., 2019 [32]. In order to predict a change in these variables, a regression approach could include prediction of a future z-normalized test score [33], which is often the raw test score corrected for age, sex, and education level [33,34]. For classification, a popular categorization is defining “stable” and “declining” subjects [35], although wording can differ. In Filippi et al., 2013, for example, the authors defined cognitive worsening as an increase in impaired tests in a cognitive test battery over time, where impairment was defined as having a z-normalized test score below two [36]. Colato et al., 2021 defined worsening as a 10% decline of the SDMT score over time [37]. We furthermore note that practice effects can occur in cognitive tests over time [38]. To correct for this, a “reliable change index” was used in Eijlers et al., 2018 [35] and Cacciaguerra et al., 2019 [39]. Lastly, up until now, outcomes were all objective measures of cognition, while subjective, or self-reported measures also receive attention as outcomes for MS prognosis [40].

### 4.2. Which Features to Take into Account?

To be able to predict a future change in the variable of interest, the input of the machine learning model should receive careful consideration. Except when modelling on raw input data, learning should occur on features that are deemed informative towards the outcome to be predicted. To this end, we can use prognostic biomarkers, which were intensively studied in recent decades. However, although evidence on cognitive prognostic biomarkers exists, comprehensive reviews on the topic were mainly made for physical deterioration. We refer to reviews that summarize prognostic biomarkers for different modalities; demographics [41], clinical information [41], CNS imaging [42,43,44,45], molecular information [9], and neurophysiology [45]. Yet, there appears to be an overlap between physical and cognitive prognostic biomarkers. Although it is beyond the scope of this review to provide a summary of cognitive biomarkers, we refer to studies that identified cognitive prognostic biomarkers for different modalities such as demographics [35,46,47], clinical information [35,46,47], MRI [35,46,48], optical coherence tomography (OCT) [49], molecular information [50], and neurophysiology [51].

In analogy with the previous question on outcomes, subjective measures might also be informative for the prediction of disease course, such as patient-reported outcomes (PRO) [52]. Specifically for cognitive prognosis, features such as subjective cognitive impairment [47] and perceived ability to concentrate [53] were found to be informative.

### 4.3. On Which Time-Frame Should Predictions Be Made?

The literature usually makes a distinction between short-term and long-term prognosis. No clear cut-off between them has been reported, and this most probably depends on the clinical query that is addressed. Short-term prognosis is by far the most intensively studied [23,25,28], while Zhao et al., 2017 presented a longer-term predictive model of 5 years [24]. Yperman et al., 2020 stated that their rationale for a 2-year timeframe was based on maximizing the number of observations in the dataset [28]. Data availability is highly likely to hinder the field in performing longer-term predictions using machine learning, but studies investigating prognostic biomarkers for long-term disability already show promising results [36,46].

### 4.4. Which Machine Learning Algorithm to Use?

Given the heterogeneity in methodology throughout the literature, it is too preliminary to make firm statements regarding the superiority of one algorithm over another when considering performance. However, a second consideration is model complexity; linear models could underfit data, but are easy to interpret and familiar for clinicians. As illustrated by Sidey-Gibbons et al., 2019 [54], algorithms capable of handling increased complexity are in general harder to understand. This is, for example, the case for (deep) neural networks, which are often regarded as black box models [54].

### 4.5. How to Assess a Machine Learning Model?

**Figure 2:**
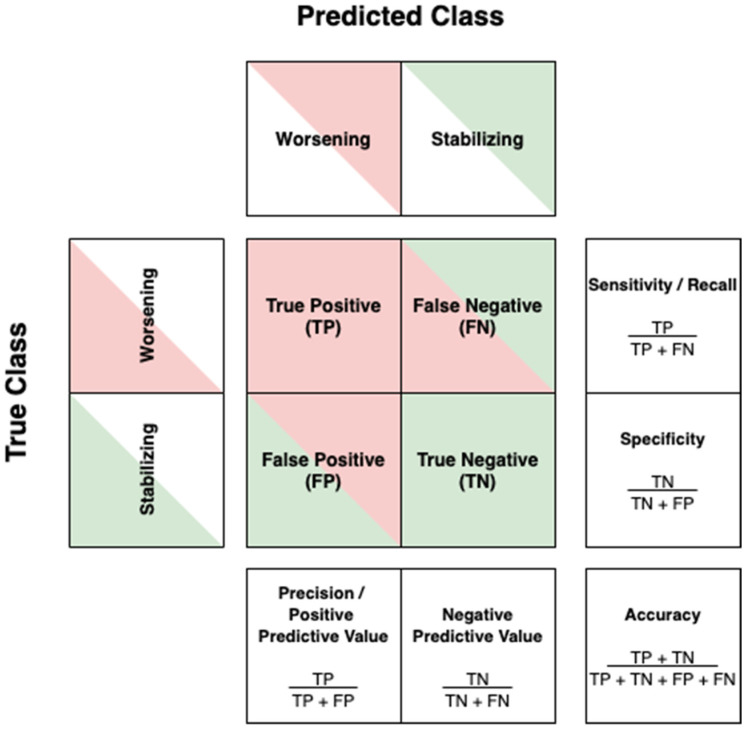
The confusion matrix and its derived metrics.

Classifications will typically yield a so-called confusion matrix. In the case of a dichotomous endpoint, the confusion matrix is a 2 × 2 matrix with one axis indicating the true group labels and the other axis the predicted group labels. An example using the labels “worsening” versus “stabilizing” is illustrated in Figure 2, along with the metrics that can be calculated from this matrix. The different metrics allow us to study model performance from different perspectives. When looking at the confusion matrix of Figure 2, low sensitivity will leave worsening patients undetected, which causes neurologists to falsely assume that their patient is stabilizing. Withholding treatment—while this is in fact justified—will potentially endanger the patient’s well-being. The opposite is true when we encounter low specificity; patients that do not worsen over time might receive treatment, while administration could potentially induce adverse events in their case.

Regarding regression performance, the most intuitive metric is the mean absolute error (MAE); it represents how much on average the predicted value deviates from the true value, while making abstraction of whether this is an under- or overestimation. The main difference with related metrics such as the normalized root-mean-square error (NRMSE, RMSE [55], MSE) is that MAE retains the unit of the outcome variable. Other performance metrics include the correlation between the true and predicted outcome [56], the variance explained by the input features (R^2^) [55], and the Akaike Information Criterion [55].

### 4.6. How Should Authors Report the Performance of Their Machine Learning Model?

Solid interpretation and comparability of models stands or falls with how papers describe their methodology and performance. As discussed in the previous subsection, different performance metrics give different insights in model performance. Although the importance of a given metric mostly depends on the domain context, it is essential to not only report scores such as accuracy, sensitivity, and specificity, but also present the raw confusion matrix in classification problems. For regression, a 2-column data frame with the predicted and true ground-truth label allows the calculation of measures such as the MAE, NRMSE, RMSE, MSE, and correlation coefficient. Providing such results in publications (e.g., in supplementary materials [27]) would be a leap forward in terms of reproducible research, while the anonymity of subjects remains assured.

The benefit is twofold. Firstly, the readership of machine learning papers can extract other metrics that they are interested in. Secondly, it would also allow future reviews on machine learning models to move beyond a narrative design. In systematic reviews for example, meta-analysis and meta-regression allows for the quantitative synthetization of data, which is possible since randomized controlled trials (RCTs) are strongly recommended to adhere to the CONSORT statement [57], guiding RCT authors towards correct, transparent, and complete reports. Although the CONSORT statement is not applicable to machine learning research, another statement in the “Enhancing the QUAlity and Transparency Of health Research” (EQUATOR, https://www.equator-network.org/, accessed on 8 December 2021) network is in fact applicable: the “Transparent Reporting of a multivariable prediction model for Individual Prognosis Or Diagnosis” (TRIPOD) statement [58].

### 4.7. When Is a Model Ready for Clinical Practice?

In order to introduce a predictive model in clinical practice, extensive technical validations and clinical performance evaluations are required, which should be complemented by ethical considerations and risk analysis. There needs to be maximal transparency towards the model’s performance, so that regulators and clinicians can establish whether its error is acceptable in view of the potential risks to patients. However, when do we judge a machine learning model to be performant enough to be translated into a clinical decision support system (CDSS) [59]? In this regard, a first milestone is whether it performs better than random, but in a second phase, it should compare favorably against other potentially simpler models, such as decision rules based on single prognostic biomarkers. Among other factors, model complexity might influence the trust of clinicians in artificial intelligence (AI) [60]. Furthermore, it would be informative to know how the machine’s prognostic accuracy relates to the accuracy of human prediction, in this case of the neurologist. Although the literature on the latter is scarce, we identified one paper on the accuracy of decoding cognitive impairment in MS, albeit cross-sectional [61]. The authors found the accuracy to be comparable to chance, and highlighted the need for improved cognitive screening [61]. In order to benchmark how a model would perform in similar conditions to actual clinical practice, study designs should directly compare the prognostic accuracy of medical professionals without and with the assistance of the considered CDSS. A typical scenario involves comparing whether the CDSS helps bridging the gap between medical professionals with different levels of experience. For instance, an ongoing trial investigates prognostic accuracy of junior and senior doctors in the domain of traumatic brain injury [62].

We note that although some models might be complex, several methods exist to enhance clinicians’ trust. In Tousignant et al., 2019 [25], deep learning, which is currently one of the most complex machine learning algorithms, was used to predict worsening in EDSS from MR images. The authors used a two-step process to gain the clinician’s trust, namely by quantifying the model’s confidence in its own predictions, and verifying whether the model is correct when it is confident [25]. We note that a whole field of research, i.e., explainable AI (XAI), is dedicated to, among other things, augmenting user trust [63].

### 4.8. Which Data to Use?

To address this question, we refer back to the section on “Specific Pitfalls for Medical Data”, where we discussed study versus real-world data, single- versus multi-center data, and dealing with multiple visits of the same subject.

## 5. State-of-the-Art ML-Powered Cognitive Prognostic Models

Literature in the field is scarce. This was confirmed by a PubMed search using the following search strategy: “(((multiple sclerosis[MeSH Terms]) OR (multiple sclerosis)) AND ((cognit*) OR (cognition[MeSH Terms]))) AND ((((machine learning[MeSH Terms]) OR (machine learning)) OR (artificial intelligence[MeSH Terms])) OR (artificial intelligence))”, which was run on 3 December 2021, and yielded 39 records. Among those, we identified two studies that used machine learning for cognitive prognosis; Kiiski et al., 2018 [56] and Lopez-Soley et al., 2021 [64]. Kiiski et al., 2018 used supervised machine learning on different combinations of multimodal data, including demographic, clinical, and electro-encephalography (EEG) data to predict short-term: (1) overall cognitive performance and (2) performance on information processing speed on a combined sample of persons with MS and healthy controls [56]. Lopez-Soley et al., 2021 also used multimodal data, including demographic, clinical, and MRI data, to predict short-term future cognitive impairment. This section is dedicated to the lessons that can be learned from their efforts.

Kiiski et al., 2018

First of all, the use of multimodal data is a good choice in light of the complex nature of MS and the identification of prognostic biomarkers in multiple domains. Moreover, the previous literature in the field of epilepsy established the superiority of multimodal data compared to using a single modality for machine learning predictions [65]. Secondly, the authors chose to z-normalize results for each neuropsychological test based on the mean and standard deviation (SD) of their sample, and use composite z-scores (average z-score of multiple tests) as the ground-truth label. A composite score was created for general cognitive functioning and one for information processing speed. Although transformation of raw test results allows comparison between, and aggregation of, different tests, the downside is in terms of clinical interpretation; clinicians have a reference frame for the original test results, whereas they do not for z-scores. Thirdly, the authors extracted over 1000 spatiotemporal features, whereas only 78 observations were used. This can be considered a large imbalance with a risk for overfitting, especially when considering the aforementioned rule of thumb of at least 10 times as many observations as features. The risk for overfitting might however have been reduced for several reasons:

- Using the “Elastic Net” [66] as learning algorithm. This is in essence a linear regression approach, but it uses regularization, which is the addition of constraints to the learning process to increase a model’s generalizability. Specifically, it uses a combination of L1 (Lasso) and L2 (Ridge) regularization, which both tend to shrink large feature weights, whereas Lasso additionally tends to remove unimportant features from the model [66]. The low complexity of linear regression combined with regularization might have increased generalizability;
- Using cross-validation (CV), which is a technique that allows the use of data for both training and validation purposes by training multiple models. If no CV were used, only one model would have been created on a part of the data, whereas validation would happen on the remaining data. Since this is a balance between few data for training (risk for a poorly trained model) and few data for validation (risk for a poor evaluation of the model), CV is a useful technique to minimize both risks.

Fourthly, we previously mentioned the importance of benchmarking to obtain a reference frame for the quality of the prediction. For this, the authors created a “null model” by shuffling the ground-truth values across subjects before starting the learning phase. According to the authors, this provides an intuition in the “level of optimism inherent in the model” [56]. Lastly, we highlighted that using a combined sample of persons with MS and healthy controls increases sample size, but it obfuscates a clear interpretation of its value for prognosis in MS. Their best-performing model for general cognitive functioning included all available data modalities and yielded a mean cross-validated correlation of 0.44.

Lopez-Soley et al., 2021

Opposed to the regression approach of Kiiski et al., 2018 [56], Lopez-Soley et al., 2021 used a classification approach to predict future global- and domain-specific cognitive impairment [64]. The risk of overfitting was reduced by using Lasso regularization during logistic regression, 10-fold cross-validation, and retaining as much data as possible by imputing missing values.

Since cognitively impaired subjects were underrepresented for global cognition and all cognitive domains, it can be considered good practice that the authors used the “balanced accuracy” ((sensitivity + specificity)/2) to assess model performance across cognitive domains. The difference with accuracy (cfr. Figure 2) can be clarified with an example. Say that in a dataset, 20 persons with MS experience cognitive decline, and 80 do not. If the model correctly classifies 70 of the 80 stabilizing subjects, but only 5 of the 20 worsening subjects, the model achieves an accuracy of (70 + 5)/100 = 75%. The balanced accuracy, however is ((5/20) + (70/80))/2 = 56.25%. Hence, the balanced accuracy might be adopted for datasets that are unbalanced. For perfectly balanced datasets, accuracy and balanced accuracy yield the same value. Based on this metric, the authors reported the best performances for verbal memory (79%) and for attention/information processing speed (73%).

We note that by reporting the true class distribution, the authors greatly contributed to the interpretation of their result, as any evaluation metric can now be assessed with respect to that reference frame.

Overall, both studies yielded valuable intuition in the future design of machine learning studies for cognitive prognosis in MS. Despite the fact that predictions were obtained on a sample of both persons with MS and healthy controls in Kiiski et al., 2018 [56], predictive performances of both studies might serve as benchmarks for evaluating future studies in the field.

## 6. ML Trends and Opportunities for Prognostic Modelling in MS

Although studies dealing with prognostic modelling of cognitive evolution in MS are scarce, we see several interesting avenues for ML-driven prognostication in MS. We will discuss alternative approaches for prognostication, the simulation of treatment response and solutions to scarcity of longitudinal data.

### 6.1. Alternative Approaches for Prognostication

Hybrid predictions. Tacchella et al., 2018 introduced the proof-of-concept “hybrid predictions” [29] in the field of MS prognosis. The authors hypothesized that the discrepancy in “reasoning” between human and machine could in fact complement each other. Their results showed that the aggregation of human (medical students) and machine predictions consistently outperformed any of the single instances in predicting the conversion from relapsing–remitting to secondary progressive MS [29]. Besides performance, the fact that human intelligence is still involved in predictions could reassure clinicians that models do not solely rely on artificial intelligence, since they also rely on expert knowledge that algorithms might not be able to learn.

Digital twin. The field of machine learning for MS prognostication is mutually geared towards augmenting personalized care with personalized predictions. Since the prediction relies on the profile of a subject in terms of multimodal data, a subject can also be represented in a digital way, i.e., a digital twin. The concept of a digital twin was discussed elaborately in a recent review by Voigt et al., 2021, highlighting its potential to predict future disease course and simulate treatment effect [67].

### 6.2. Simulation of Treatment Response

Up until now, studies on prognostication mostly focused on predicting the natural course of multiple sclerosis. In our view, this is a necessary step to subsequently be able to predict, in a personalized way, how this natural course changes by administering certain treatment such as disease-modifying therapy (DMT). Although such estimates might be even more challenging, Pruenza et al., 2019 aimed to predict individual responses to 14 different DMTs [68]. The authors assigned a score per DMT that represented the likelihood of no disability progression in case of administration of the DMT [68]. Beyond a research effort, the authors created a tool that allows users to predict treatment response in new patients [68].

### 6.3. Solutions to Scarcity of Longitudinal Data

Transfer learning. A potential solution to scarcity of longitudinal data is to mitigate the necessity of building a model from scratch by using a robustly trained model from another domain, mostly related to the domain of interest. To this end, neural networks are typically used. Since the network’s weights are meaningful to solve a related task, they could be used as initialization for the task of interest, updating the weights using a smaller dataset. For example, Nanni et al., 2020 used pretrained networks (trained on the ImageNet database [69]) to classify pictures of everyday objects (number of pictures in the order of millions), for prognostic purposes in Alzheimer’s disease (number of MR images in the order of hundreds) [70].

Federated learning. For various reasons, data sharing in medical sciences remains delicate [71], which might explain why efforts in ML-powered prognostication remain largely single-center, extracting data from a single central database (centralized approach). However, an increasing number of studies [72,73] prove that machine learning can also occur in a decentralized way, i.e., by federated learning, meaning that data remain at their original location, while still being used for machine learning in a remote location.

Continual learning. In continual learning, an AI is not trained once, but evolves over time by augmenting performance along with the ever-going supply of novel data. The implications of this technique in medical sciences are nicely discussed in Lee et al., 2020 [74].

## 7. Conclusions

Machine learning is a rising concept in light of clinical decision support systems and personalized medicine and could boost the quest to find a suitable predictive algorithm for prognosis in MS. Investigations should however also address cognitive deterioration, and authors should be maximally transparent in reporting their results to allow comparison in the field. In doing so, clinical decision support systems using machine learning to predict future cognitive deterioration in MS could become a reality in clinical practice, providing the best possible personalized care for persons with MS.

## 8. Key Messages

- Machine learning is capable of handling multimodal data and could predict disease course on an individual level;
- The literature on cognitive prognosis using machine learning in MS is scarce. Future studies on machine learning for prognosis in MS should not overlook cognitive deterioration;
- Recommendations for the design of studies on machine learning for cognitive prognosis are proposed;
- Researchers should aim to share as many results as possible to allow benchmarking, solid interpretation, and comparison in the field, for example, by sharing raw predictions;
- Several trends in machine learning could overcome current roadblocks in ML-powered prognostic modelling in MS, such as scarcity of longitudinal data.

## Figures and Tables

**Table 1:** Supervised machine learning techniques exemplified for binary classification and univariate regression. For ease of interpretation, all examples use a low-dimensional feature space. However, the same principle holds when adding features towards higher-dimensional feature spaces.

Method	Description	Visualization
Logistic Regression	Logistic regression identifies the optimal sigmoid curve between the two labels to be predicted, yielding a probability of belonging to either of the two groups. In the illustration: the probability that a person will worsen or stabilize over time.	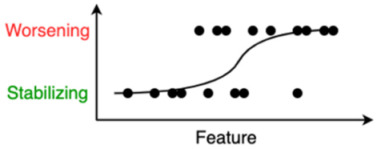
Decision Tree	A decision tree is a sequence of decisions that are made on certain criteria. The last leaves of the tree indicate one of the class labels that are to be predicted.	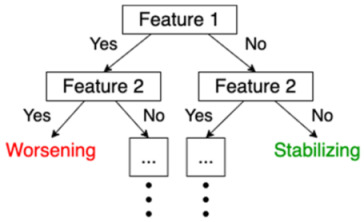
Random Forest	This is an example of “ensemble learning”, meaning that learning, and thus the resulting model, relies on multiple learning strategies, aiming to average the error out [15]. In this case, a random forest consists of multiple decision trees, mitigating the bias introduced by relying on one single decision tree. The ultimate prediction of a random forest classifier is the majority vote of the predictions of the individual decision trees in the random forest.	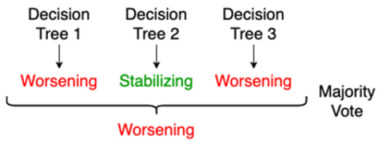
SVM	In case of two features, a support vector machine (SVM) tries to find a line or a curve that separates the two classes of interest. It does so by maximizing the distance between the line and the data-points on both sides of the line, thus maximally separating both classes.	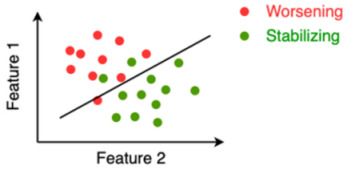
ANN	An artificial neural network (ANN) was inspired by the neural network of the brain and consists of nodes (weights) and edges that connect the nodes. Input data in either raw form or a feature representation enters the ANN on the left (input layer) and gets modified by the ANN in the hidden layers using the nodes’ weights learned during the training phase, so that the input is optimally reshaped, or “mapped”, to the endpoint that needs to be predicted on the right (output layer).	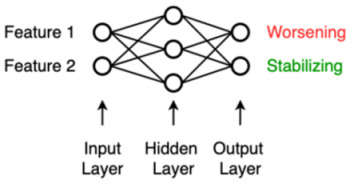
Linear Regression	Linear regression is a technique in which the weight of every input feature is learned, which is multiplied with their respective feature and summed together with the so-called “bias” (also a learned weight but not associated to a feature, i.e., a constant), yielding a prediction that minimizes the error with the ground-truth. In the 2D case, this is the line that minimizes the sum of the squared vertical distances of individual points to the regression line. The learned weights in this case are the slope (β_1_) and intercept (β_0_, bias) of the line.	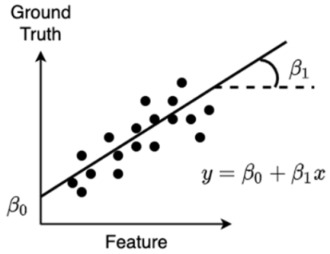

## Data Availability

Not applicable.

## References

[1] Winquist R.J., Kwong A., Ramachandran R., Jain J. (2007). The complex etiology of multiple sclerosis. Biochem. Pharmacol..

[2] Brown F.S., Glasmacher S.A., Kearns P.K.A., MacDougall N., Hunt D., Connick P., Chandran S. (2020). Systematic review of prediction models in relapsing remitting multiple sclerosis. PLoS ONE.

[3] Weinstock-Guttman B., Sormani M.P., Repovic P. (2021). Predicting Long-term Disability in Multiple Sclerosis: A Narrative Review of Current Evidence and Future Directions. Int. J. MS Care.

[4] Seccia R., Romano S., Salvetti M., Crisanti A., Palagi L., Grassi F. (2021). Machine Learning Use for Prognostic Purposes in Multiple Sclerosis. Life.

[5] Moazami F., Lefevre-Utile A., Papaloukas C., Soumelis V. (2021). Machine Learning Approaches in Study of Multiple Sclerosis Disease Through Magnetic Resonance Images. Front. Immunol..

[6] Lejbkowicz I., Caspi O., Miller A. (2012). Participatory medicine and patient empowerment towards personalized healthcare in multiple sclerosis. Expert Rev. Neurother..

[7] Reich D.S., Lucchinetti C.F., Calabresi P.A. (2018). Multiple Sclerosis. N. Engl. J. Med..

[8] Kister I., Bacon T.E., Chamot E., Salter A.R., Cutter G.R., Kalina J.T., Herbert J. (2013). Natural History of Multiple Sclerosis Symptoms. Int. J. MS Care.

[9] Ziemssen T., Akgün K., Brück W. (2019). Molecular biomarkers in multiple sclerosis. J. Neuroinflamm..

[10] Macías Islas M., Ciampi E. (2019). Assessment and Impact of Cognitive Impairment in Multiple Sclerosis: An Overview. Biomedicines.

[11] Clemens L., Langdon D. (2018). How does cognition relate to employment in multiple sclerosis? A systematic review. Mult. Scler. Relat. Disord..

[12] Kavaliunas A., Karrenbauer V.D., Gyllensten H., Manouchehrinia A., Glaser A., Olsson T., Alexanderson K., Hillert J. (2019). Cognitive function is a major determinant of income among multiple sclerosis patients in Sweden acting independently from physical disability. Mult. Scler..

[13] Definition of Machine Learning. https://www.lexico.com/definition/machine_learning.

[14] Lecun Y., Bengio Y., Hinton G. (2015). Deep learning. Nature.

[15] Polikar R., Zhang C., Ma Y. (2012). Ensemble Machine Learning.

[16] Hastie T., Tibshirani R., Friedman J. (2009). The Elements of Statistical Learning.

[17] Alibakshi A. (2018). Strategies to develop robust neural network models: Prediction of flash point as a case study. Anal. Chim. Acta..

[18] DeVries T., Taylor G.W. (2017). Dataset Augmentation in Feature Space. arXiv.

[19] Goodfellow I.J., Pouget-Abadie J., Mirza M., Xu B., Warde-Farley D., Ozair S., Courville A., Bengio Y. (2014). Generative Adversarial Networks. Commun. ACM.

[20] Remeseiro B., Bolon-Canedo V. (2019). A review of feature selection methods in medical applications. Comput. Biol. Med..

[21] Jolliffe I.T., Cadima J. (2016). Principal component analysis: A review and recent developments. Philos. Trans. R. Soc. A Math. Phys. Eng. Sci..

[22] Bejarano B., Bianco M., Gonzalez-Moron D., Sepulcre J., Goñi J., Arcocha J., Soto O., Carro U.D., Comi G., Leocani L. (2011). Computational classifiers for predicting the short-term course of Multiple sclerosis. BMC Neurol..

[23] Yoo Y., Tang L.W., Brosch T., Li D.K.B., Metz L., Traboulsee A., Tam R. (2016). Deep Learning of Brain Lesion Patterns for Predicting Future Disease Activity in Patients with Early Symptoms of Multiple Sclerosis. Lecture Notes in Computer Science (Including Subseries Lecture Notes in Artificial Intelligence and Lecture Notes in Bioinformatics).

[24] Zhao Y., Healy B.C., Rotstein D., Guttmann C.R.G., Bakshi R., Weiner H.L., Brodley C.E., Chitnis T. (2017). Exploration of machine learning techniques in predicting multiple sclerosis disease course. PLoS ONE.

[25] Tousignant A., Lemaître P., Precup D., Arnold D.L., Arbel T. Prediction of Disease Progression in Multiple Sclerosis Patients using Deep Learning Analysis of MRI Data. Proceedings of the 2nd International Conference on Medical Imaging with Deep Learning.

[26] Buda M., Maki A., Mazurowski M.A. (2018). A systematic study of the class imbalance problem in convolutional neural networks. Neural Netw..

[27] Seccia R., Gammelli D., Dominici F., Romano S., Landi A.C., Salvetti M., Tacchella A., Zaccaria A., Crisanti A., Grassi F. (2020). Considering patient clinical history impacts performance of machine learning models in predicting course of multiple sclerosis. PLoS ONE.

[28] Yperman J., Becker T., Valkenborg D., Popescu V., Hellings N., Van Wijmeersch B., Peeters L.M. (2020). Machine learning analysis of motor evoked potential time series to predict disability progression in multiple sclerosis. BMC Neurol..

[29] Tacchella A., Romano S., Ferraldeschi M., Salvetti M., Zaccaria A., Crisanti A., Grassi F. (2018). Collaboration between a human group and artificial intelligence can improve prediction of multiple sclerosis course: A proof-of-principle study. F1000Research.

[30] Kourou K., Exarchos T.P., Exarchos K.P., Karamouzis M.V., Fotiadis D.I. (2015). Machine learning applications in cancer prognosis and prediction. Comput. Struct. Biotechnol. J..

[31] Sumowski J.F., Benedict R., Enzinger C., Filippi M., Geurts J.J., Hamalainen P., Hulst H., Inglese M., Leavitt V.M., Rocca M.A. (2018). Cognition in multiple sclerosis: State of the field and priorities for the future. Neurology.

[32] Oreja-Guevara C., Ayuso Blanco T., Brieva Ruiz L., Hernández Pérez M.Á., Meca-Lallana V., Ramió-Torrentà L. (2019). Cognitive Dysfunctions and Assessments in Multiple Sclerosis. Front. Neurol..

[33] Ouellette R., Bergendal Å., Shams S., Martola J., Mainero C., Kristoffersen Wiberg M., Fredrikson S., Granberg T. (2018). Lesion accumulation is predictive of long-term cognitive decline in multiple sclerosis. Mult. Scler. Relat. Disord..

[34] Costers L., Gielen J., Eelen P.L., Van Schependom J., Laton J., Van Remoortel A., Vanzeir E., Van Wijmeersch B., Seeldrayers P., Haelewyck M.C. (2017). Does including the full CVLT-II and BVMT-R improve BICAMS? Evidence from a Belgian (Dutch) validation study. Mult. Scler. Relat. Disord..

[35] Eijlers A.J.C., van Geest Q., Dekker I., Steenwijk M.D., Meijer K.A., Hulst H.E., Barkhof F., Uitdehaag B.M.J., Schoonheim M.M., Geurts J.J.G. (2018). Predicting cognitive decline in multiple sclerosis: A 5-year follow-up study. Brain.

[36] Filippi M., Preziosa P., Copetti M., Riccitelli G., Horsfield M.A., Martinelli V., Comi G., Rocca M.A. (2013). Gray matter damage predicts the accumulation of disability 13 years later in MS. Neurology.

[37] Colato E., Stutters J., Tur C., Narayanan S., Arnold D.L., Gandini Wheeler-Kingshott C.A.M., Barkhof F., Ciccarelli O., Chard D.T., Eshaghi A. (2021). Predicting disability progression and cognitive worsening in multiple sclerosis using patterns of grey matter volumes. J. Neurol. Neurosurg. Psychiatry.

[38] Portaccio E., Goretti B., Zipoli V., Iudice A., Pina D.D., Malentacchi G.M., Sabatini S., Annunziata P., Falcini M., Mazzoni M. (2010). Reliability, practice effects, and change indices for Raos brief repeatable battery. Mult. Scler..

[39] Cacciaguerra L., Pagani E., Mesaros S., Dackovic J., Dujmovic-Basuroski I., Drulovic J., Valsasina P., Filippi M., Rocca M.A. (2019). Dynamic volumetric changes of hippocampal subfields in clinically isolated syndrome patients: A 2-year MRI study. Mult. Scler. J..

[40] Beier M., Amtmann D., Ehde D.M. (2015). Beyond depression: Predictors of self-reported cognitive function in adults living with MS. Rehabil. Psychol..

[41] Degenhardt A., Ramagopalan S.V., Scalfari A., Ebers G.C. (2009). Clinical prognostic factors in multiple sclerosis: A natural history review. Nat. Rev. Neurol..

[42] Louapre C., Bodini B., Lubetzki C., Freeman L., Stankoff B. (2017). Imaging markers of multiple sclerosis prognosis. Curr. Opin. Neurol..

[43] Kearney H., Miller D.H., Ciccarelli O. (2015). Spinal cord MRI in multiple sclerosis—diagnostic, prognostic and clinical value. Nat. Rev. Neurol..

[44] Davda N., Tallantyre E., Robertson N.P. (2019). Early MRI predictors of prognosis in multiple sclerosis. J. Neurol..

[45] Leocani L., Rocca M.A., Comi G. (2016). MRI and neurophysiological measures to predict course, disability and treatment response in multiple sclerosis. Curr. Opin. Neurol..

[46] Dekker I., Eijlers A.J.C., Popescu V., Balk L.J., Vrenken H., Wattjes M.P., Uitdehaag B.M.J., Killestein J., Geurts J.J.G., Barkhof F. (2019). Predicting clinical progression in multiple sclerosis after 6 and 12 years. Eur. J. Neurol..

[47] Fuchs T.A., Wojcik C., Wilding G.E., Pol J., Dwyer M.G., Weinstock-Guttman B., Zivadinov R., Benedict R.H. (2020). Trait Conscientiousness predicts rate of longitudinal SDMT decline in multiple sclerosis. Mult. Scler. J..

[48] Hildesheim F.E., Benedict R.H.B., Zivadinov R., Dwyer M.G., Fuchs T., Jakimovski D., Weinstock-Guttman B., Bergsland N. (2021). Nucleus basalis of Meynert damage and cognition in patients with multiple sclerosis. J. Neurol..

[49] Bsteh G., Hegen H., Teuchner B., Amprosi M., Berek K., Ladstätter F., Wurth S., Auer M., Di Pauli F., Deisenhammer F. (2019). Peripapillary retinal nerve fibre layer as measured by optical coherence tomography is a prognostic biomarker not only for physical but also for cognitive disability progression in multiple sclerosis. Mult. Scler. J..

[50] Gold S.M., Raji A., Huitinga I., Wiedemann K., Schulz K.-H., Heesen C. (2005). Hypothalamo–pituitary–adrenal axis activity predicts disease progression in multiple sclerosis. J. Neuroimmunol..

[51] Nauta I.M., Kulik S.D., Breedt L.C., Eijlers A.J., Strijbis E.M., Bertens D., Tewarie P., Hillebrand A., Stam C.J., Uitdehaag B.M. (2021). Functional brain network organization measured with magnetoencephalography predicts cognitive decline in multiple sclerosis. Mult. Scler. J..

[52] Brichetto G., Bragadin M.M., Fiorini S., Battaglia M.A., Konrad G., Ponzio M., Pedullà L., Verri A., Barla A., Tacchino A. (2020). The hidden information in patient-reported outcomes and clinician-assessed outcomes: Multiple sclerosis as a proof of concept of a machine learning approach. Neurol. Sci..

[53] De Groot V., Beckerman H., Uitdehaag B.M., Hintzen R.Q., Minneboo A., Heymans M.W., Lankhorst G.J., Polman C.H., Bouter L.M. (2009). Physical and Cognitive Functioning After 3 Years Can Be Predicted Using Information From the Diagnostic Process in Recently Diagnosed Multiple Sclerosis. Arch. Phys. Med. Rehabil..

[54] Sidey-Gibbons J.A.M., Sidey-Gibbons C.J. (2019). Machine learning in medicine: A practical introduction. BMC Med. Res. Methodol..

[55] Kuceyeski A., Monohan E., Morris E., Fujimoto K., Vargas W., Gauthier S.A. (2018). Baseline biomarkers of connectome disruption and atrophy predict future processing speed in early multiple sclerosis. NeuroImage Clin..

[56] Kiiski H., Jollans L., Donnchadha S.Ó., Nolan H., Lonergan R., Kelly S., O’Brien M.C., Kinsella K., Bramham J., Burke T. (2018). Machine Learning EEG to Predict Cognitive Functioning and Processing Speed Over a 2-Year Period in Multiple Sclerosis Patients and Controls. Brain Topogr..

[57] Schulz K.F. (2010). CONSORT 2010 Statement: Updated Guidelines for Reporting Parallel Group Randomized Trials. Ann. Intern. Med..

[58] Moons K.G.M., Altman D.G., Reitsma J.B., Ioannidis J.P.A., Macaskill P., Steyerberg E.W., Vickers A.J., Ransohoff D.F., Collins G.S. (2015). Transparent reporting of a multivariable prediction model for individual prognosis or diagnosis (TRIPOD): Explanation and elaboration. Ann. Intern. Med..

[59] Sutton R.T., Pincock D., Baumgart D.C., Sadowski D.C., Fedorak R.N., Kroeker K.I. (2020). An overview of clinical decision support systems: Benefits, risks, and strategies for success. NPJ Digit. Med..

[60] Asan O., Bayrak A.E., Choudhury A. (2020). Artificial Intelligence and Human Trust in Healthcare: Focus on Clinicians. J. Med. Internet Res..

[61] Romero K., Shammi P., Feinstein A. (2015). Neurologists’ accuracy in predicting cognitive impairment in multiple sclerosis. Mult. Scler. Relat. Disord..

[62] Comparison of the Accuracy of the Neurological Prognosis at 6 Months of Traumatic Brain Injury Between Junior and Senior Doctors—Full Text View—ClinicalTrials.gov. https://clinicaltrials.gov/ct2/show/NCT04810039.

[63] Gunning D., Stefik M., Choi J., Miller T., Stumpf S., Yang G.-Z. (2019). XAI—Explainable artificial intelligence. Sci. Robot..

[64] Lopez-Soley E., Martinez-Heras E., Andorra M., Solanes A., Radua J., Montejo C., Alba-Arbalat S., Sola-Valls N., Pulido-Valdeolivas I., Sepulveda M. (2021). Dynamics and Predictors of Cognitive Impairment along the Disease Course in Multiple Sclerosis. J. Pers. Med..

[65] Memarian N., Kim S., Dewar S., Engel J., Staba R.J. (2015). Multimodal data and machine learning for surgery outcome prediction in complicated cases of mesial temporal lobe epilepsy. Comput. Biol. Med..

[66] Zou H., Hastie T. (2005). Regularization and variable selection via the elastic net. J. R. Stat. Soc. Ser. B (Stat. Methodol.).

[67] Voigt I., Inojosa H., Dillenseger A., Haase R., Akgün K., Ziemssen T. (2021). Digital Twins for Multiple Sclerosis. Front. Immunol..

[68] Pruenza C., Solano M.T., Diaz J., Arroyo-Gonzalez R., Izquierdo G. (2019). Model for Prediction of Progression in Multiple Sclerosis. Int. J. Interact. Multimed. Artif. Intell..

[69] Deng J., Dong W., Socher R., Li L.-J., Li K., Li F.-F. ImageNet: A large-scale hierarchical image database. Proceedings of the 2009 IEEE Conference on Computer Vision and Pattern Recognition.

[70] Nanni L., Interlenghi M., Brahnam S., Salvatore C., Papa S., Nemni R., Castiglioni I., Initiative T.A.D.N. (2020). Comparison of Transfer Learning and Conventional Machine Learning Applied to Structural Brain MRI for the Early Diagnosis and Prognosis of Alzheimer’s Disease. Front. Neurol..

[71] Van Panhuis W.G., Paul P., Emerson C., Grefenstette J., Wilder R., Herbst A.J., Heymann D., Burke D.S. (2014). A systematic review of barriers to data sharing in public health. BMC Public Health.

[72] Brisimi T.S., Chen R., Mela T., Olshevsky A., Paschalidis I.C., Shi W. (2018). Federated learning of predictive models from federated Electronic Health Records. Int. J. Med. Inform..

[73] Aledhari M., Razzak R., Parizi R.M., Saeed F. (2020). Federated Learning: A Survey on Enabling Technologies, Protocols, and Applications. IEEE Access Pract. Innov. Open Solut..

[74] Lee C.S., Lee A.Y. (2020). Clinical applications of continual learning machine learning. Lancet Digit. Health.

